# Retraction: Effects of the Forecasting Methods, Precipitation Character, and Satellite Resolution on the Predictability of Short-Term Quantitative Precipitation Nowcasting (QPN) from a Geostationary Satellite

**DOI:** 10.1371/journal.pone.0196228

**Published:** 2018-04-17

**Authors:** The PLOS ONE Editors

After publication, concerns were raised about overlap in content published in this *PLOS ONE* article [1] and an article published in *Journal of Hydrology* [2]. There is overlap in the research objectives and conclusions, text overlap in the Methods and Results, and overlap between the following figures and tables:

- *PLOS ONE* Fig 1 (a1, a2, b1, b2, h1, h2) appears to duplicate *J*. *Hydrology* Fig 2(a-f)
- *PLOS ONE* Fig 2 panels are highly similar to *J*. *Hydrology* Fig 4 (b, f)
- *PLOS ONE* Fig 3a, b are highly similar to *J*. *Hydrology* Fig 6b, a
- *PLOS ONE* Tables 1 and 3 are highly similar to *J*. *Hydrology* Tables 1 and 3, respectively

The *Journal of Hydrology* article was not mentioned at the time of submission to *PLOS ONE*, and was not cited or discussed in the *PLOS ONE* article.

A member of *PLOS ONE*’s Editorial Board confirmed the overlap in content between the two articles and noted that while there are some additions to the *PLOS ONE* article relative to what was reported in *Journal of Hydrology*, these differences are not sufficient to represent a distinct publication.

In light of these concerns, the *PLOS ONE* Editors retract this article.

YL, DGX, and ZLL do not agree with retraction and stand by the article’s conclusion. WJ could not be reached.
